# The Role of Polyunsaturated Fatty Acids in Osteoarthritis: Insights from a Mendelian Randomization Study

**DOI:** 10.3390/nu15224787

**Published:** 2023-11-15

**Authors:** Xuefei Li, Zhengjie Lu, Yongjian Qi, Biao Chen, Bin Li

**Affiliations:** 1 Department of Pathology, Union Hospital, Tongji Medical College, Huazhong University of Science and Technology, Wuhan 430022, China; lixfwurm@163.com; 2 Division of Joint Surgery and Sports Medicine, Department of Orthopedic Surgery, Zhongnan Hospital of Wuhan University, Wuhan 430071, Chinachenbiao20030701@163.com (B.C.); 3 Department of Spine Surgery and Musculoskeletal Tumor, Department of Orthopedic Surgery, Zhongnan Hospital of Wuhan University, Wuhan 430071, China

**Keywords:** polyunsaturated fatty acids, omega-3 fatty acids, omega-6 fatty acids, osteoarthritis, Mendelian randomization

## Abstract

The prior observational research on the impact of polyunsaturated fatty acid (PUFA) supplementation on osteoarthritis (OA) patients had yielded inclusive outcomes. This study utilized the Mendelian randomization (MR) approach to explore potential causal relationships between PUFAs and OA. The MR study was performed using GWAS summary statistics for PUFAs, encompassing omega-3 and omega-6 fatty acids, and for knee OA (KOA) and hip OA (HOA). The primary inverse-variance-weighted (IVW) method and two supplementary MR approaches were used to establish robust causality. Heterogeneity and horizontal pleiotropy were assessed using Cochrane’s Q and MR-Egger intercept tests. Additionally, a range of sensitivity analyses were conducted to strengthen the precision and reliability of the results. The IVW method indicated a potential genetic association between omega-3 fatty acids and KOA risk (odd ratio (OR) = 0.94, 95% confidence interval (CI): 0.89–1.00, *p* = 0.048). No significant correlation was found between omega-3 levels and HOA. Moreover, genetically predicted higher levels of omega-6 fatty acids were associated with a decreased risk of KOA (OR = 0. 93, 95% CI: 0.86–1.00, *p* = 0.041) and HOA (OR = 0.89, 95% CI: 0.82–0.96; *p* = 0.003). The MR-Egger intercept evaluation showed no horizontal pleiotropy affecting the MR analysis (all *p*  >  0.05). Our findings supported the causal relationship between PUFAs and OA susceptibility and offered a novel insight that high omega-6 fatty acids may reduce the risk of KOA and HOA. These results underscore the importance of maintaining optimal levels of PUFAs, particularly omega-6 fatty acids, in individuals with a genetic predisposition to OA. Future research is necessary to validate these findings and elucidate the underlying mechanisms involved.

## 1. Introduction

Osteoarthritis (OA), a chronic degenerative joint disease, primarily targets the knee and hip joints. The global prevalence of this condition is estimated to exceed 500 million individuals, accounting for approximately 7% of the world’s population [1]. Projections by the United Nations suggest that this percentage is expected to rise to 15–20% by the year 2050 [2]. In light of the high occurrence of OA, the search for effective treatments beyond end-stage surgeries such as total joint arthroplasty continues. Additionally, the increasing expenses related to surgical interventions [3] and the significant impact of OA on individuals, economies, and societies mean that it is imperative to investigate alternative and complementary strategies that can alleviate symptoms and improve functional outcomes. While various medications have been employed in OA patients with an improved understanding of its pathogenesis, none have demonstrated significant efficacy in symptom relief [4,5]. Given these medications’ potential long-term adverse effects in OA patients, there is a pressing need to identify alternative therapeutic agents.

Nutritional interventions targeting osteoarthritis (OA) have gained attention, as including anti-inflammatory nutrients holds potential benefits [6]. Polyunsaturated fatty acids (PUFAs), including omega-3 and omega-6 fatty acids, are obtained from various sources and can be incorporated into routine diet to maintain health. Omega-3 fatty acids, abundant in fatty fish, seafood, cereals, seeds, nuts, and vegetables, are widely consumed; however, Western diets are rich in omega-6 fatty acids [7]. Omega-3 fatty acids have demonstrated efficacy in benefiting OA patients [8]. Conversely, omega-6 fatty acids were previously believed to stimulate the secretion of pro-inflammatory cytokines [9]. These two pivotal nutritional bioactive compounds are often regarded as having divergent physiological functions [10]. However, the results of previous randomized controlled trials (RCTs) assessing the efficacy of omega-3 fatty acid supplementation in individuals with OA have shown inconsistent findings. While certain studies have indicated that additional supplementation of omega-3 fatty acids may alleviate arthritis pain in individuals with OA [11,12,13], others have not corroborated these findings [14,15]. Notably, no RCTs to date have examined the impact of omega-6 fatty acids on OA. 

As an alternative research method, Mendelian randomization (MR) has emerged as a valuable analytical approach that utilizes genetic variants, specifically single-nucleotide polymorphisms (SNPs), as instrumental variables to emulate the random allocation commonly seen in randomized controlled trials (RCTs) [16,17]. In situations where reliable RCTs are lacking, or initiating new RCTs may be impractical, MR serves as an ideal strategy to elucidate causal relationships between exposures and outcomes [18]. Moreover, MR effectively avoids reverse causality, as the formation of genotypes precedes disease onset and remains unaffected by disease progression [19,20]. Notably, to our knowledge, no MR studies have been conducted to explore the relationship between PUFAs and OA. Therefore, this study employed the MR method to investigate potential causal associations between PUFAs and the risk of developing OA. We aimed for the outcomes of this investigation to enhance our comprehension of the underlying pathophysiology of OA and offer substantial evidence for establishing effective treatment and prevention strategies in clinical practice.

## 2. Materials and Methods

### 2.1. Study Design

**Figure 1:**
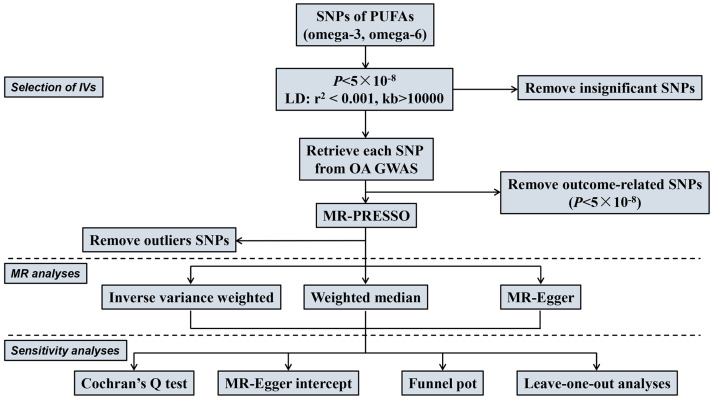
Study flame chart of the MR study evaluating the causality between PUFAs and osteoarthritis. SNPs, single-nucleotide polymorphisms; PUFAs, polyunsaturated fatty acids; IVs, instrumental variables; LD, linkage disequilibrium; OA, osteoarthritis; IVW, inverse-variance-weighted; GWAS, genome-wide association study; MR, Mendelian randomization.

In this study, we employed an MR approach utilizing summary statistics derived from genome-wide association studies (GWASs) to examine the associations between polyunsaturated fatty acids (PUFAs), specifically omega-3 fatty acids and omega-6 fatty acids, and osteoarthritis (OA), including knee OA (KOA) and hip OA (HOA). To ensure the robustness of our MR analysis, we adhered to three fundamental assumptions: (i) the selected genetic variants should exhibit significant associations with the exposure of interest; (ii) the genetic variants employed should not exert any influence on the outcome, except through the chosen exposure; and (iii) the genetic variants should not be correlated with any confounding factors that may impact the relationship between the exposure and the outcome. The detailed study design is illustrated in Figure 1.

### 2.2. Data Sources

The SNP summary data associated with polyunsaturated fatty acids (PUFAs) were obtained from the Nightingale Health UK Biobank Initiative, which provided access to information on both omega-3 and omega-6 fatty acids for our investigation [20]. The genetic instrumental variables for omega-3 and omega-6 were sourced from the Metabolic Biomarkers in the UK Biobank study—the GWAS associated with omega-3 and omega-6 fatty acids involved 114,999 European individuals [20]. The UK Biobank initiative aims to explore the genetic and environmental factors contributing to disease, with a participant pool of over 500,000 individuals of European descent. For this project, the circulating concentrations of omega-3 and omega-6 fatty acids were assessed in randomly selected EDTA plasma samples using a targeted high-throughput nuclear magnetic resonance (NMR) metabolomics platform provided by Nightingale Health Ltd. The initial sample collection comprised 121,577 samples, with duplicates and observations not meeting the quality control criteria in the non-fasting plasma samples collected at baseline being excluded. This platform’s measurement technology and applications for epidemiological studies have been previously evaluated [21,22].

Summary-level data for the SNP associated with KOA and HOA were obtained from the UK Biobank study [23]. The datasets of KOA and HOA comprised many individuals of European ancestry, respectively, including 403,124 individuals and 393,873 individuals. The OA cases were sourced from the Arthritis Research UK Osteoarthritis Genetics (arcOGEN) project, which comprises unrelated individuals of European descent with knee or hip osteoarthritis from the arcOGEN Consortium. These cases were identified based on either clinical evidence of disease necessitating joint replacement or radiographic evidence of disease with a Kellgren–Lawrence grade of 2 or higher. The controls were obtained from the United Kingdom Household Longitudinal Study (UKHLS), a longitudinal panel survey representing 40,000 households across England, Scotland, Wales, and Northern Ireland and representative of the UK population. The genetic associations between polyunsaturated fatty acids (PUFAs) and OA are presented in Table 1.

### 2.3. Selection of Instrumental Variables (IVs)

When selecting IVs for our MR analysis, we strictly followed three well-established assumptions: (i) IVs should exhibit strong associations with the exposure of interest; (ii) IVs should be independent of any confounding factors; and (iii) IVs should solely influence the outcomes through the chosen exposure, without having any direct associations with the outcomes themselves [24]. We first applied a genome-wide significance threshold (*p* < 5 × 10^−8^) to initiate the IV screening process for our MR study. Subsequently, we implemented SNP pruning within a window size of 10,000 kb, ensuring that the linkage disequilibrium (LD) between SNPs remained below the threshold of r^2^ < 0.001. Furthermore, the selected SNPs related to Omega-3 and Omega-6 fatty acids were retrieved from the GWAS data of the outcomes (KOA and HOA), and any SNPs significantly associated with the outcomes (*p* < 5 × 10^−8^) were excluded. In addition, we carefully assessed the IVs selected through the above process for any indications of weak instrument bias by using the F-statistic [25,26]. To mitigate bias stemming from weak IVs, we exclusively retained IVs with an F value surpassing 10, while those with an F value of less than 10 were discarded due to weak instrument bias [27].

### 2.4. MR Analysis

The causal effects of PUFAs (omega-3 and omega-6 fatty acids) on knee and hip OA were systematically examined using the R package TwoSample MR (version 0.5.7), adhering to established methodologies [28]. The primary technique for estimating causal effects was the random-effects inverse-variance weighted (IVW) method [29]. Alongside the IVW method, we utilized two additional MR methods (MR-Egger and weighted median) to evaluate causal associations. The IVW method operates under the assumption that all SNPs included in the analysis are valid instrumental variables [30]. The weighted median approach presupposes that a minimum of 50% of genetic variants are valid, satisfying the three fundamental assumptions. It is applicable in situations where the majority of IVs do not demonstrate horizontal pleiotropy [31]. In contrast, the MR-Egger regression method assumes that more than 50% of genetic variants are invalid (do not adhere to the three fundamental assumptions), which may result in a slightly lower estimation accuracy being achieved with this approach [32,33]. The MR estimates were presented as beta or odds ratios (ORs) with 95% confidence intervals (CIs). The Bonferroni correction is a method for selecting a threshold *p*-value due to the assumption that every variant tested is independent of the rest. The statistical calculation via the Bonferroni correction method is *p* = 0.05/(number of exposures × number of outcomes), widely used to evaluate the significance of causal relationship between exposures and outcomes in MR studies [34,35,36]. To account for multiple tests in our MR study, we applied a Bonferroni correction with 4 tests, setting a significance threshold of *p*  <  0.0125 (0.05/2 × 2). 

Significant associations (*p*  < 0.05) before but not after Bonferroni correction (*p*  <  0.005) were considered as suggestive association results [24]. If the IVW method result is significant and no pleiotropy is detected, despite the insignificant results of other methods, it can be considered a positive result, provided that the beta values of the other methods are in the same direction [37].

We conducted a series of sensitivity analyses in our study, including Cochran’s Q test, MR-Egger intercept, funnel plots, and leave-one-out analyses, so as to explore potential heterogeneity and pleiotropy and ensure the robustness of the results. Heterogeneity was assessed via implementing IVW and MR-Egger regressions, with Cochran’s Q statistic utilized for evaluation [38]. If the *p* values of Cochran’s Q test for IVW and MR-Egger are greater than 0.05, it does not indicate significant heterogeneity. To ascertain the absence of pleiotropy in our MR results, we examined the intercept in the MR-Egger regression, with a *p* value exceeding 0.05, indicating no presence of pleiotropy [39]. To evaluate directional pleiotropy, we visually analyzed MR-Egger intercepts and funnel plots. A leave-one-SNP-out analysis was conducted to further investigate causal relationships, to prevent the MR analysis results from being driven by a single SNP [40]. 

## 3. Results

### 3.1. Causal Effects of Omega-3 Fatty Acids on KOA and HOA

**Figure 2:**
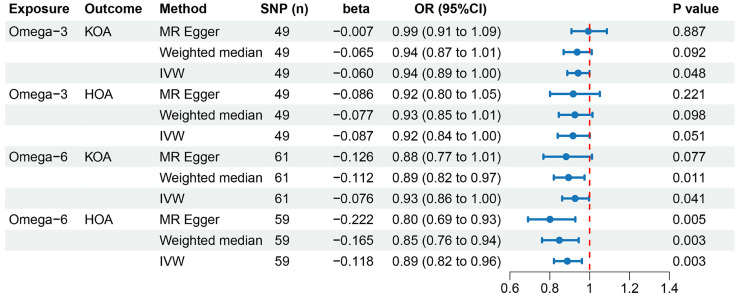
Estimation of the causal relationship between PUFAs and OA using different MR methods. SNPs, single-nucleotide polymorphisms; KOA, knee osteoarthritis; HOA, hip osteoarthritis; IVW, inverse-variance-weighted; PUFAs, polyunsaturated fatty acids; MR, Mendelian randomization.

Figure 2 depicts the MR methods to ascertain the causal relationships between omega-3 fatty acids and OA. A total of 49 LD-independent and suitable IVs were chosen from GWASs for KOA and HOA (Supplementary Table S1). The primary IVW analysis suggested a potential association between omega-3 fatty acids and a reduced risk of KOA (*p*  =  0.048). A 1 SD increase in omega-3 fatty acids corresponded to an OR and 95% (CI) for KOA of OR = 0.94 (95% CI: 0.89–1.00). However, other MR methods, including MR-Egger (OR = 0.99, 95% CI: 0.91–1.09; *p* = 0.887) and weighted median (OR = 0.94, 95% CI: 0.87–1.01; *p* = 0.092), indicated a consistent but non-significant direction (Figure 2). Given the significant results of the IVW and the consistent direction of beta values from other methods, this can be interpreted as a positive result [22,37]. Consequently, omega-3 fatty acids were deemed to have a causal association with a decreased risk of KOA. However, no significant associations were observed between omega-3 fatty acids and HOA risk (*p* = 0.051).

**Figure 3:**
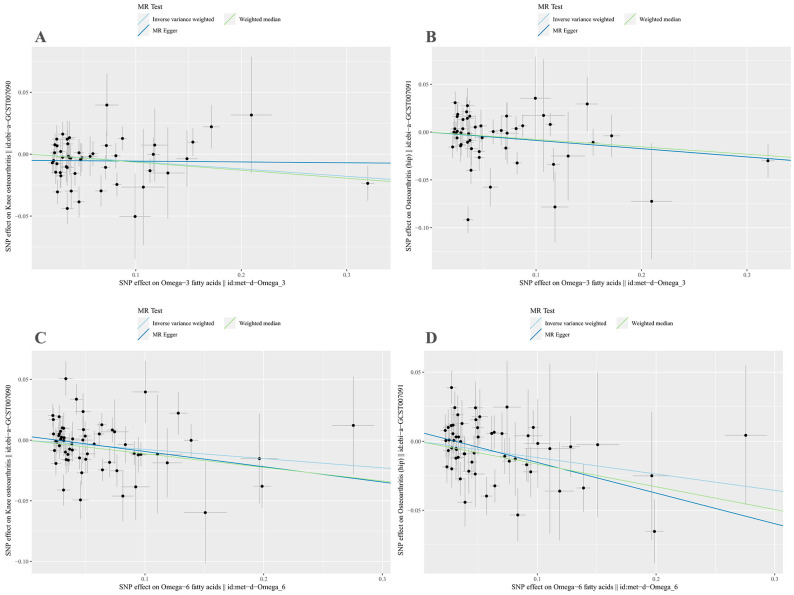
Scatter plots of genetic correlations of omega-3/omega-6 fatty acids and KOA/HOA using different MR methods. (**A**) Scatter plot of genetic correlations of omega-3 fatty acids with KOA. (**B**) Scatter plot of genetic correlations of omega-3 fatty acids with HOA. (**C**) Scatter plot of genetic correlations of omega-6 fatty acids with KOA. (**D**) Scatter plot of genetic correlations of omega-6 fatty acids with HOA. SNPs, single-nucleotide polymorphisms; KOA, knee osteoarthritis; HOA, hip osteoarthritis; MR, Mendelian randomization.

**Figure 4:**
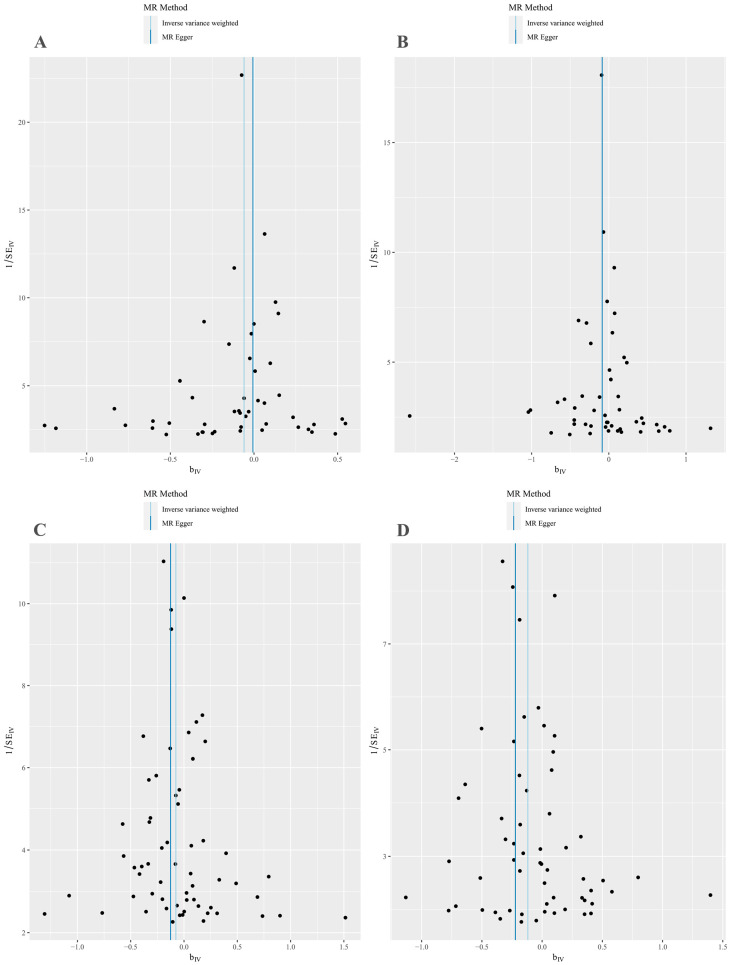
Funnel plots of SNPs associated with omega-3/omega-6 fatty acids and KOA/HOA. (**A**) Funnel plot for omega-3 fatty acids with KOA. (**B**) Funnel plot for omega-3 fatty acids with HOA. (**C**) Funnel plot for omega-6 fatty acids with KOA. (**D**) Funnel plot for omega-6 fatty acids with HOA. SNPs, single-nucleotide polymorphisms; KOA, knee osteoarthritis; HOA, hip osteoarthritis.

**Figure 5:**
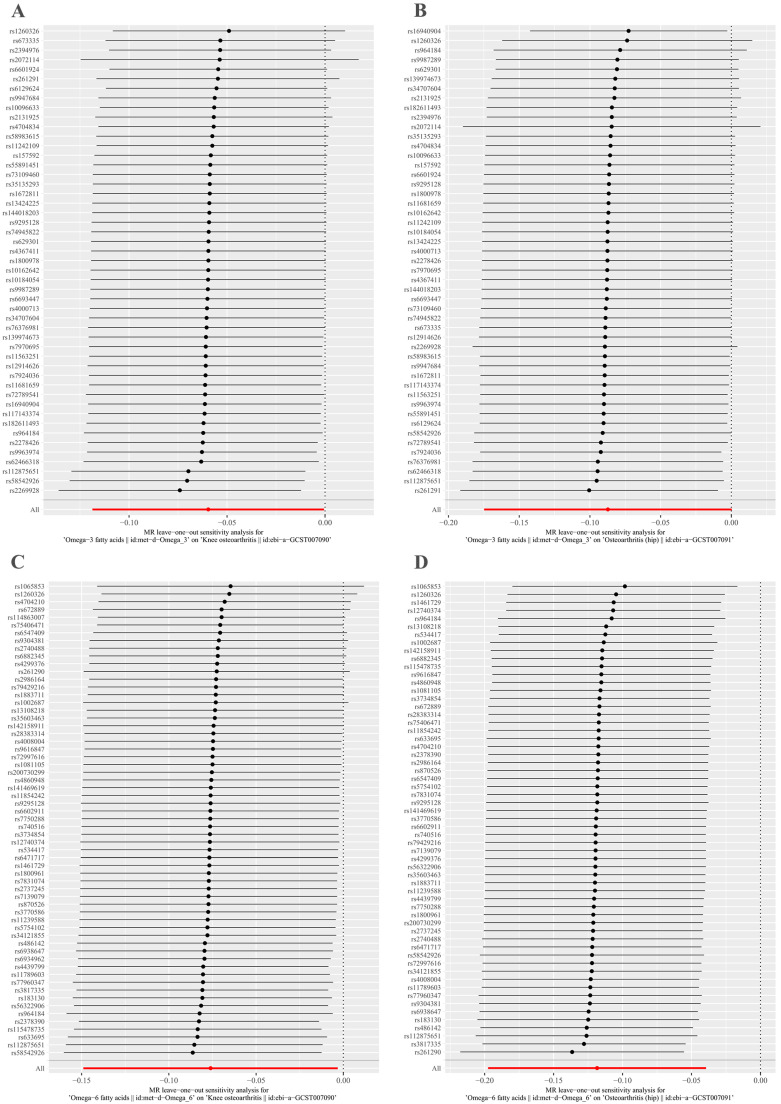
Leave-one-out plots to visualize the causal effects of omega-3/omega-6 fatty acids on KOA/HOA. (**A**) Leave-one-out analysis for omega-3 fatty acids with KOA. (**B**) Leave-one-out analysis for omega-3 fatty acids with HOA. (**C**) Leave-one-out analysis for omega-6 fatty acids with KOA. (**D**) Leave-one-out analysis for omega-6 fatty acids with HOA. KOA, knee osteoarthritis; HOA, hip osteoarthritis.

MR-Egger regression and IVW analyses were conducted to assess heterogeneity. Heterogeneity was observed in the MR analyses of omega-3 fatty acids for KOA (MR-Egger: *p* = 0.009; IVW: *p* = 0.005) and HOA (MR-Egger: *p* = 1.498 × 10^−6^; IVW: *p* = 2.304 × 10^−6^) (Table 2). Given the heterogeneity, using random-effects IVW estimation was appropriate in evaluating causality under these circumstances [41]. Nevertheless, the IVW results consistently supported the causal relationship between omega-3 fatty acids and KOA risk (Figure 2). Additionally, the MR-Egger intercept tests indicated no horizontal pleiotropy in any of the analyses, as all *p* values exceeded 0.05 (Table 2). The scatter plot illustrated the estimated impact of SNPs on omega-3 fatty acids and KOA/HOA (Figure 3A,B). Furthermore, the leave-one-out analysis demonstrated that no outlier instrumental variables significantly influenced the overall results (Figure 4A,B). The funnel plot of the omega-3 fatty acids and KOA/HOA analysis indicated no apparent horizontal pleiotropy, as the variation in effect size around the point estimate was symmetrical (Figure 5A,B).

### 3.2. Causal Effects of Omega-6 on KOA and HOA

Figure 2 depicts the MR analyses examining the causal relationships between omega-6 fatty acids and OA risk. In total, 61 and 59 LD-independent and suitable IVs were chosen from GWASs for KOA and HOA (Supplementary Tables S2 and S3). The IVW method suggested that genetically predicted omega-6 fatty acids might be associated with a reduced risk of KOA (OR = 0.93, 95% CI: 0.86–1.00; *p* = 0.041). The weighted median analysis yielded similar significant results (OR = 0.89, 95% CI: 0.82–0.97; *p* = 0.011), while the MR-Egger analysis showed consistent but non-significant results (OR = 0.88, 95% CI: 0.77–1.01; *p* = 0.077). Given the significant results of the IVW and the consistent direction of beta values from the other two methods [22,37], these findings suggest that omega-3 fatty acids may have a causal association with a decreased risk of KOA. Simultaneously, our IVW method provided evidence of genetically predicted omega-6 fatty acids being associated with a reduced risk of HOA (OR = 0.89, 95% CI: 0.82–0.96; *p* = 0.003). Significant results were observed using both the MR-Egger (OR = 0.80, 95% CI: 0.69–0.93; *p* = 0.005) and weighted median (OR = 0.85, 95% CI: 0.76–0.94; *p* = 0.003) methods, aligning with the findings obtained through the IVW approach.

Considering the potential heterogeneity observed in the MR analyses of omega-6 for KOA (MR-Egger: *p* = 5.993 × 10^−5^; IVW: *p*  =  6.037 × 10^−5^) and HOA (MR-Egger: *p* = 0.052; IVW: *p* = 0.035) (Table 2), we employed the random-effects IVW method to further support the causal relationship between omega-6 and KOA/HOA (Figure 2). No evidence of horizontal pleiotropy was found in this analysis, as indicated by both intercepts having *p* values greater than 0.05 (Table 2). The scatter plots displaying the individual causal estimates were presented (Figure 3C,D). The results remained consistent even after removing one SNP, as shown by the leave-one-out test (Figure 4C,D). The funnel plots illustrated the analyses of omega-6 and KOA/HOA (Figure 5C,D).

## 4. Discussion

In this MR study, we employed extensive GWAS summary data to examine the potential causal association between PUFAs and the risk of OA in individuals of European ancestry. Our analysis involved a sizable cohort of individuals with European heritage, comprising 114,999 individuals for PUFAs and OA, 450,243 individuals for KOA, and 256,523 individuals for HOA. The findings demonstrated that genetically predicted omega-3 fatty acids were linked to a decreased risk of KOA, while no significant association was observed with HOA. Furthermore, our results revealed an innovative association between genetically predicted omega-6 fatty acids and a decreased risk of both KOA and HOA. Significantly, this study endeavours to investigate the causal association between PUFAs and OA risk using an MR method. By elucidating the impact of PUFAs on OA risk, our findings effectively bridge a critical research gap and present promising prospects for personalized treatment strategies.

Omega-3 fatty acids encompass alpha linolenic acid (ALA), eicosapentaenoic acid (EPA), and docosahexaenoic acid (DHA). ALA is primarily sourced from flaxseed oil, tahini, certain nuts, and seeds, while EPA and DHA are predominantly present in oily marine fish and certain seaweed. Unlike plant cells, the human body lacks the necessary enzymes to synthesize ALA, making dietary intake the sole means of obtaining this fatty acid. However, the accumulation of ALA at significant levels is uncommon, even when consumed in relatively high dietary quantities. This limited accumulation is primarily attributed to the β-oxidation of dietary ALA within the mitochondria hampering its conversion to EPA and DHA, which occurs at a minimal rate of less than 1% [42,43]. The level of EPA and DHA in the tissues and cells can be increased through their direct dietary consumption. Therefore, omega-3 fatty acids, mainly referring to EPA and DHA, should be consumed in the diet from fish oil or functional foods fortified with them [44]. Omega-3 fatty acids have been proposed as potential therapeutic agents for individuals with OA due to their ability to mitigate the systemic inflammatory response and create an environment that counteracts cartilage degradation [8]. However, previous observational studies investigating the efficacy of omega-3 fatty acids in OA patients have produced inconsistent outcomes. In one study, 86 participants diagnosed with OA were randomly allocated to receive either a cod liver oil supplement (786 mg EPA) or a placebo (olive oil) in conjunction with their existing non-steroidal anti-inflammatory drug regimen for 24 weeks. The utilization of cod liver oil did not yield significant differences in reported pain or disability compared to the placebo group [45]. Similarly, another study revealed that omega-3 fatty acids did not substantially improve functional scores [14]. Additionally, an early meta-analysis suggested that marine oil supplementation with a high content of EPA and DHA could potentially alleviate arthritis-related pain. However, it is worth noting that most of the studies included in the analysis focused on patients with rheumatoid arthritis. When subgroup analysis was conducted on five studies involving individuals with OA, the potential effectiveness of marine oil in managing arthritis pain was not substantiated [46].

Nevertheless, there is also substantiated evidence supporting the utilization of omega-3 fatty acids in the management of OA. One RCT trial that enrolled 202 patients indicated that low-dose fish oil containing EPA and DHA led to greater improvement in pain and function scores in KOA at 2 years [47]. Recently, Stonehouse et al. reported that krill oil containing EPA and DHA could improve pain, stiffness, and physical function for individuals with mild-to-moderate KOA [12]. Moreover, a recent systematic review and meta-analysis, encompassing data from nine RCTs involving 2070 individuals with OA, demonstrated that supplementation with omega-3 fatty acids resulted in a significant reduction in arthritis pain and improvement in joint function [48]. Our study outcomes establish a causal association between omega-3 fatty acids and the risk of KOA, consistent with recent meta-analysis findings [48]. The potential mechanisms underlying the beneficial effects of omega-3 fatty acids on KOA are multifactorial, which might include the inhibition of inflammatory markers such as interleukin-1 beta (IL-1β) and inducible nitric oxide synthase (iNOS), the suppression of metalloproteinase 13 expression and chondrocyte apoptosis, and the restraining of bone remodelling and vessel formation within the osteochondral unit [46,49,50]. It is imperative to acknowledge that EPA and DHA exhibit distinct effects. EPA can convert into DHA within the liver and primarily functions to reduce cellular inflammation. It exerts anti-inflammatory actions by inhibiting the enzyme delta-5-desaturase (D5D), responsible for synthesizing arachidonic acid (AA), an omega-6 fatty acid known to mediate cellular inflammation. Moreover, EPA competes with AA for the enzyme phospholipase A2, essential for liberating AA from membrane phospholipids. This synergetic interaction aids in diminishing the generation of inflammatory eicosanoids [44]. Nevertheless, the literature on the optimal concentration ratio of EPA and DHA for individuals with OA is still limited. Further research in this domain is warranted.

Omega-6 fatty acids mainly comprise AA and linoleic acid (LA). AA is a precursor to various potent pro-inflammatory mediators, such as prostaglandins and leukotrienes, which have been extensively studied. Biochemically, AA derived from LA leads to the production of pro-inflammatory molecules. Consequently, an increased intake of omega-6 fatty acids, either AA or its precursor LA, is widely believed to promote inflammation [51]. However, the human studies available to date do not support this assumption. This discrepancy may be attributed to the previously mentioned low in vivo conversion rate of dietary LA to AA [52]. Moreover, the available data essentially refute the idea that high intakes of omega-6 fatty acids result in systemic inflammation. A notable example is a cross-sectional study that scrutinized the dietary levels of PUFAs in 364 individuals with confirmed cardiovascular disease. The findings revealed that omega-6 fatty acids had an inverse association with c-reactive protein (CRP) levels and IL-1β [53]. Another epidemiological study reported that individuals in the highest quintiles of omega-6 fatty acids exhibited lower concentrations of CRP and reduced incidences of cardiovascular disease, cancer, and all-cause mortality [54]. Several additional studies have consistently showcased an inverse correlation between circulating levels of omega-6 fatty acids and inflammatory biomarkers [55,56,57]. A comprehensive meta-analysis concluded that randomized controlled intervention studies fail to furnish evidence supporting a causal link between heightened consumption of LA and elevated concentrations of inflammatory markers [58]. Up to date, no RCTs or MR studies have been undertaken to elucidate the impact of omega-6 fatty acids on susceptibility to OA. Our study effectively addressed this research void by presenting compelling evidence of the advantageous effects of omega-6 fatty acids on KOA and HOA. It has been reported that some oxylipins produced by AA exhibit pro-inflammatory properties, while other eicosanoids derived from AA show anti-inflammatory activities [51]. However, the administration of AA (which is challenging due to its limited availability) at relatively high doses has not altered circulating inflammation biomarkers, at least in healthy volunteers [59,60]. Hence, these beneficial effects of omega-6 fatty acids might be attributed to the inherent anti-inflammatory properties associated with LA [52]. One intervention trial showed that an increased intake of LA does not increase circulating concentrations of AA and AA-derived lipid mediators [61]. Therefore, the supplementation of LA may be a more practical and promising strategy in the future. 

The implications of these findings are of significant clinical importance. Our results unequivocally demonstrate that maintaining optimal levels of PUFAs, specifically omega-6 fatty acids, may effectively mitigate the risk of both KOA and HOA. These findings align with the outcomes of a prospective study, wherein 2029 participants diagnosed with KOA exhibited less joint space loss over 4 years when they consumed higher quantities of PUFAs [62]. Consequently, healthcare professionals may consider an augmented intake of PUFA-rich foods or supplements for individuals afflicted with OA, particularly those with a genetic predisposition. However, it is essential to note that a global study has reported suboptimal omega-6 fatty acids intake worldwide [63]. According to the Global Burden of Disease 2017 group, a lack of adequate dietary intake of omega-6 fatty acids is considered one of the critical factors associated with diet-related cardiovascular disease [64]. Given that nutritional recommendations and guidelines play a crucial role in shaping health policies, it is essential to carefully analyze and evaluate the quantity and quality of dietary fats within the context of a healthy diet. Seed oils, mainly vegetable oils, are rich sources of omega-6 fatty acids, with LA comprising more than 50% of their lipid content. Other significant sources of LA include nuts, while lower levels can be found in whole grains, legumes, non-ruminant meats, eggs, and dairy products [52]. The recommended limits for total and saturated fat intake, as established by authoritative societies, typically fall below 30–35% and 10% of the total energy, respectively. The recommended intake for total PUFAs, including omega-3 and omega-6 fatty acids, typically falls within 5% to 10% of energy intake. However, recommendations for omega-6 fatty acids, especially LA, are less consistent across societies, with most suggesting intakes between 2.5% and 5% of total energy [52]. Nonetheless, it is vital to conduct further research to substantiate the clinical viability of these recommendations.

This MR research features four major noteworthy strengths. Firstly, this study employed genetic variants as IVs to evaluate the causal impacts of PUFAs (omega-3 and omega-6 fatty acids) on the risk of knee and hip OA. Through the utilization of the MR approach, concerns regarding confounding factors and reverse causation, commonly encountered in conventional observational investigations, were mitigated to the greatest extent. Secondly, the study made use of large-scale genomic data obtained from the UK Biobank, thereby bolstering the universality and robustness of our innovative discoveries. Thirdly, the utilization of publicly accessible datasets and open-source software in this study contributes to the transparency and repeatability of our MR research. Lastly, the MR analysis strategy facilitates the estimation of causal effect sizes, which carries both theoretical and practical implications for sensible clinical or public health decision making. 

Several considerations should be acknowledged regarding our study. Firstly, it is worth noting that the dataset utilized in our GWAS analysis solely consisted of individuals of European descent. Consequently, the generalizability of our findings to other ethnic groups is limited, hindering the exploration of cultural diversity. Secondly, heterogeneity was observed in our results. However, we effectively addressed this issue by employing the random-effect IVW method as our primary analytical approach, successfully controlling for pooled heterogeneity. Thirdly, it is essential to highlight that, due to the constraints imposed by GWAS summary data, a stratified analysis based on crucial factors such as age and gender could not be conducted. Therefore, future studies should encompass more extensive and diverse populations, incorporating individuals with varied ancestries and cultural backgrounds. Additionally, these studies should adopt a combination of observational and genetic approaches to further investigate the causal effects of PUFAs on OA, while concurrently considering stratified factors, such as age and gender.

## 5. Conclusions

To sum up, our study has provided compelling evidence of a genetic causal relationship between PUFAs and OA risk, as demonstrated through the two-sample MR analysis. Specifically, our findings indicate that increased levels of omega-3 fatty acids were associated with a decreased risk of KOA. Intriguingly, our results challenged the traditional belief regarding the detrimental impact of omega-6 fatty acids on OA, as higher levels of omega-6 fatty acids decreased the risk of both KOA and HOA. These findings suggest that targeted dietary interventions aimed at modulating omega-6 fatty acid levels might serve as an effective preventive strategy for mitigating the burden of OA. However, it is essential to note that further research is indispensable to validate these findings, preferably through large-scale longitudinal studies or RCTs. Additionally, exploring the underlying mechanisms behind these observed associations could provide valuable insights into the pathogenesis of OA.

## Figures and Tables

**Table 1:** Details of the GWAS summary-level data.

Trait	Dataset	Sample Size	GWAS ID	Population
Omega-3	UK Biobank	114,999	met-d-Omega_3	European
Omega-6	UK Biobank	114,999	met-d-Omega_6	European
KOA	UK Biobank	403,124	ebi-a-GCST007090	European
HOA	UK Biobank	393,873	ebi-a-GCST007091	European

GWAS, genome-wide association study; KOA, knee osteoarthritis; HOA, hip osteoarthritis.

**Table 2:** Heterogeneity and pleiotropy test of MR studies.

Exposure	Outcome	MR-Egger (*p* Value)	IVW (*p* Value)	MR-Egger Intercept (*p* Value)
Omega-3	KOA	0.009	0.005	0.131
Omega-3	HOA	1.498 × 10^−6^	2.304 × 10^−6^	0.979
Omega-6	KOA	5.993 × 10^−5^	6.037 × 10^−5^	0.404
Omega-6	HOA	0.052	0.035	0.110

MR, Mendelian randomization; KOA, knee osteoarthritis; HOA, hip osteoarthritis; IVW, inverse-variance-weighted.

## Data Availability

The summary statistics used and/or analyzed in the current study are available from the corresponding authors upon reasonable request.

## Supplementary Materials

The following supporting information can be downloaded at: https://www.mdpi.com/article/10.3390/nu15224787/s1, Table S1: Instrumental variables of Omega-3 fatty acids for knee osteoarthritis and hip osteoarthritis; Table S2: Instrumental variables of Omega-6 fatty acids for knee osteoarthritis; Table S3: Instrumental variables of Omega-6 fatty acids for hip osteoarthritis.
