# Supplementary material for: The Role of Polyunsaturated Fatty Acids in Osteoarthritis: Insights from a Mendelian Randomization Study
Source: Nutrients. 2023 Nov 15;15(22):4787. doi: 10.3390/nu15224787 (PMC10674676; doi:10.3390/nu15224787)
Supplement: Supplementary file 1 [file nutrients-15-04787-s001.zip › nutrients-2660901-supplementary.pdf]

Supplementary Tables

Table S1: Instrumental variables of Omega-3 fatty acids for knee osteoarthritis and hip osteoarthritis.

SNP	effect_allele	other_allele	beta	eaf	se	F
rs10096633	T	C	−0.039	0.124	0.006	99.027
rs10162642	A	G	−0.049	0.210	0.005	237.809
rs10184054	G	C	−0.036	0.224	0.005	138.023
rs11242109	T	G	0.024	0.479	0.004	87.590
rs112875651	A	G	−0.087	0.392	0.004	1071.996
rs11563251	T	C	0.035	0.111	0.006	72.960
rs11681659	T	C	−0.025	0.716	0.004	78.449
rs117143374	C	T	−0.037	0.142	0.006	100.665
rs1260326	C	T	−0.082	0.604	0.004	976.166
rs12914626	T	C	−0.059	0.702	0.004	440.894
rs12976395	C	G	0.029	0.505	0.004	108.121
rs13424225	T	G	0.022	0.450	0.004	73.339
rs139974673	C	T	0.118	0.026	0.013	212.235
rs144018203	C	G	0.107	0.011	0.020	68.367
rs157592	C	A	0.028	0.185	0.005	70.257
rs1672811	C	T	0.025	0.748	0.005	71.907
rs16940904	T	C	−0.036	0.227	0.005	132.706
rs1800978	G	C	−0.037	0.124	0.006	90.880
rs182611493	G	A	−0.210	0.013	0.020	286.560
rs2072114	G	A	−0.320	0.123	0.006	6746.634
rs2131925	T	G	0.071	0.646	0.004	704.494
rs2269928	G	T	−0.154	0.226	0.005	2439.528
rs2278426	T	C	−0.073	0.035	0.011	108.292
rs2394976	T	G	−0.046	0.162	0.006	175.485
rs261291	C	T	0.113	0.356	0.004	1783.202
rs34707604	C	T	0.046	0.259	0.005	221.129
rs35135293	T	C	−0.021	0.517	0.004	65.427
rs4000713	A	G	−0.029	0.295	0.004	104.402
rs4367411	T	C	0.031	0.207	0.005	83.753
rs4704834	G	A	0.029	0.644	0.004	116.729
rs5112	G	C	0.048	0.534	0.004	297.528
rs55891451	C	A	0.034	0.202	0.005	113.214
rs58542926	T	C	−0.172	0.074	0.008	1226.261
rs58983615	T	A	−0.099	0.020	0.015	111.534
rs6129624	A	G	−0.026	0.335	0.004	86.527

rs62466318	T	C	−0.072	0.204	0.005	507.465
rs629301	T	G	0.038	0.778	0.005	153.707
rs6601924	C	T	0.035	0.846	0.006	96.682
rs6693447	G	T	0.023	0.462	0.004	79.207
rs673335	C	T	−0.067	0.160	0.006	365.572
rs72789541	A	T	−0.081	0.296	0.004	826.638
rs73109460	A	G	−0.035	0.124	0.006	79.047
rs74945822	A	G	−0.048	0.143	0.006	166.085
rs76376981	C	G	−0.149	0.046	0.010	558.638
rs7924036	T	G	0.023	0.504	0.004	82.553
rs7970695	A	G	−0.025	0.621	0.004	90.944
rs9295128	T	G	−0.130	0.017	0.016	164.385
rs964184	C	G	−0.117	0.867	0.006	956.146
rs9947684	G	A	0.042	0.654	0.004	245.697
rs9963974	A	T	0.031	0.319	0.004	124.368
rs9987289	G	A	0.057	0.909	0.007	160.754

Table S2: Instrumental variables of Omega-6 fatty acids for knee osteoarthritis.

SNP	effect_allele	other_allele	beta	eaf	se	F
rs1002687	A	G	0.091	0.645	0.004	1166.075
rs1065853	T	G	−0.199	0.081	0.007	1783.454
rs1081105	C	A	0.119	0.028	0.012	231.989
rs11239588	G	A	0.029	0.268	0.005	97.879
rs112875651	A	G	−0.064	0.392	0.004	578.357
rs114863007	A	G	−0.046	0.095	0.007	108.884
rs115478735	T	A	0.042	0.183	0.005	164.256
rs11789603	T	C	0.048	0.109	0.006	136.084
rs11854242	T	C	−0.038	0.275	0.005	178.650
rs1260326	C	T	−0.064	0.604	0.004	607.683
rs12740374	T	G	−0.057	0.221	0.005	348.097
rs13108218	G	A	−0.035	0.615	0.004	177.350
rs141469619	G	A	0.111	0.010	0.021	67.295
rs142158911	A	G	−0.094	0.117	0.006	558.348
rs1461729	G	A	0.084	0.899	0.007	388.137
rs1800961	T	C	−0.074	0.030	0.012	99.886
rs183130	T	C	0.062	0.324	0.004	509.111
rs1883711	C	G	0.092	0.031	0.012	151.489
rs200730299	C	A	−0.037	0.194	0.005	113.569
rs2378390	A	G	−0.033	0.141	0.006	82.036
rs261290	C	T	−0.097	0.655	0.004	1290.077

rs2737245	T	G	−0.027	0.279	0.005	92.905
rs2740488	C	A	−0.050	0.265	0.005	299.750
rs28383314	C	T	0.039	0.623	0.004	218.170
rs2986164	A	G	−0.025	0.536	0.004	80.426
rs34121855	G	T	−0.050	0.204	0.005	243.995
rs35603463	C	T	0.034	0.567	0.005	120.765
rs3734854	A	G	0.048	0.065	0.008	84.390
rs3770586	T	C	−0.023	0.484	0.004	80.363
rs3817335	A	T	−0.028	0.351	0.004	108.508
rs4008004	A	C	0.033	0.222	0.005	114.291
rs4299376	T	G	−0.035	0.676	0.004	167.854
rs4439799	T	C	0.022	0.502	0.004	76.823
rs4704210	C	G	0.047	0.374	0.004	314.350
rs4766578	A	T	0.028	0.503	0.004	118.166
rs4860948	A	T	0.028	0.244	0.005	87.894
rs486142	A	G	0.030	0.517	0.004	141.944
rs534417	G	A	0.039	0.875	0.006	103.166
rs56322906	A	G	−0.100	0.035	0.011	209.766
rs5754102	A	C	−0.032	0.183	0.005	89.764
rs58542926	T	C	−0.128	0.074	0.008	693.781
rs633695	G	A	0.073	0.292	0.004	663.976
rs6471717	A	G	−0.029	0.663	0.004	115.170
rs6547409	T	C	−0.081	0.051	0.009	193.510
rs6602911	T	C	0.026	0.360	0.004	93.976
rs672889	G	T	0.076	0.860	0.006	433.088
rs6882345	A	G	0.045	0.633	0.004	288.401
rs6934962	T	C	0.023	0.400	0.004	77.043
rs6938647	C	A	−0.048	0.782	0.005	236.919
rs7139079	A	G	−0.030	0.593	0.004	130.578
rs72997616	A	C	−0.052	0.094	0.007	137.940
rs740516	G	C	−0.032	0.151	0.006	77.841
rs75406471	A	G	−0.031	0.155	0.006	78.924
rs7750288	G	A	0.025	0.285	0.004	77.995
rs77960347	G	A	0.276	0.013	0.018	611.776
rs7831074	G	C	0.028	0.759	0.005	76.063
rs79429216	A	G	0.151	0.013	0.018	176.327
rs870526	T	C	−0.032	0.521	0.004	155.868
rs9295128	T	G	−0.196	0.017	0.016	378.873
rs9304381	T	C	0.070	0.818	0.005	449.938
rs9616847	T	A	0.024	0.388	0.004	82.058
rs964184	C	G	−0.139	0.867	0.006	1365.365

Table S3: Instrumental variables of Omega-6 fatty acids for hip osteoarthritis.

SNP	effect_allele	other_allele	beta	eaf	se	F
rs1002687	A	G	0.091	0.645	0.004	1166.075
rs1065853	T	G	−0.199	0.081	0.007	1783.454
rs1081105	C	A	0.119	0.028	0.012	231.989
rs11239588	G	A	0.029	0.268	0.005	97.879
rs112875651	A	G	−0.064	0.392	0.004	578.357
rs115478735	T	A	0.042	0.183	0.005	164.256
rs11789603	T	C	0.048	0.109	0.006	136.084
rs11854242	T	C	−0.038	0.275	0.005	178.650
rs1260326	C	T	−0.064	0.604	0.004	607.683
rs12740374	T	G	−0.057	0.221	0.005	348.097
rs13108218	G	A	−0.035	0.615	0.004	177.350
rs141469619	G	A	0.111	0.010	0.021	67.295
rs142158911	A	G	−0.094	0.117	0.006	558.348
rs1461729	G	A	0.084	0.899	0.007	388.137
rs1800961	T	C	−0.074	0.030	0.012	99.886
rs183130	T	C	0.062	0.324	0.004	509.111
rs1883711	C	G	0.092	0.031	0.012	151.489
rs200730299	C	A	−0.037	0.194	0.005	113.569
rs2378390	A	G	−0.033	0.141	0.006	82.036
rs261290	C	T	−0.097	0.655	0.004	1290.077
rs2737245	T	G	−0.027	0.279	0.005	92.905
rs2740488	C	A	−0.050	0.265	0.005	299.750
rs28383314	C	T	0.039	0.623	0.004	218.170
rs2986164	A	G	−0.025	0.536	0.004	80.426
rs34121855	G	T	−0.050	0.204	0.005	243.995
rs35603463	C	T	0.034	0.567	0.005	120.765
rs3734854	A	G	0.048	0.065	0.008	84.390
rs3770586	T	C	−0.023	0.484	0.004	80.363
rs3817335	A	T	−0.028	0.351	0.004	108.508
rs4008004	A	C	0.033	0.222	0.005	114.291
rs4299376	T	G	−0.035	0.676	0.004	167.854
rs4439799	T	C	0.022	0.502	0.004	76.823
rs4704210	C	G	0.047	0.374	0.004	314.350
rs4766578	A	T	0.028	0.503	0.004	118.166
rs4860948	A	T	0.028	0.244	0.005	87.894
rs486142	A	G	0.030	0.517	0.004	141.944
rs534417	G	A	0.039	0.875	0.006	103.166
rs56322906	A	G	−0.100	0.035	0.011	209.766
rs5754102	A	C	−0.032	0.183	0.005	89.764
rs58542926	T	C	−0.128	0.074	0.008	693.781
rs633695	G	A	0.073	0.292	0.004	663.976

rs6471717	A	G	−0.029	0.663	0.004	115.170
rs6547409	T	C	−0.081	0.051	0.009	193.510
rs6602911	T	C	0.026	0.360	0.004	93.976
rs672889	G	T	0.076	0.860	0.006	433.088
rs6882345	A	G	0.045	0.633	0.004	288.401
rs6938647	C	A	−0.048	0.782	0.005	236.919
rs7139079	A	G	−0.030	0.593	0.004	130.578
rs72997616	A	C	−0.052	0.094	0.007	137.940
rs740516	G	C	−0.032	0.151	0.006	77.841
rs75406471	A	G	−0.031	0.155	0.006	78.924
rs7750288	G	A	0.025	0.285	0.004	77.995
rs77960347	G	A	0.276	0.013	0.018	611.776
rs7831074	G	C	0.028	0.759	0.005	76.063
rs79429216	A	G	0.151	0.013	0.018	176.327
rs870526	T	C	−0.032	0.521	0.004	155.868
rs9295128	T	G	−0.196	0.017	0.016	378.873
rs9304381	T	C	0.070	0.818	0.005	449.938
rs9616847	T	A	0.024	0.388	0.004	82.058
rs964184	C	G	−0.139	0.867	0.006	1365.365
